# Plastic biodegradation by *in vitro* environmental microorganisms and *in vivo* gut microorganisms of insects

**DOI:** 10.3389/fmicb.2022.1001750

**Published:** 2023-01-06

**Authors:** Xian-Guang Yang, Ping-Ping Wen, Yi-Fan Yang, Pan-Pan Jia, Wei-Guo Li, De-Sheng Pei

**Affiliations:** ^1^State Key Laboratory Base of Cell Differentiation and Regulation, College of Life Science, Henan Normal University, Xinxiang, China; ^2^School of Public Health, Chongqing Medical University, Chongqing, China

**Keywords:** enzyme, gut microbes, insects, invertebrate, plastic biodegradation

## Abstract

Traditional plastics, such as polyethylene (PE), polystyrene (PS), polypropylene (PP), polyvinyl chloride (PVC), polyethylene terephthalate (PET), polyurethane (PUR), and other plastic polymers, are difficult to degrade and are gradually accumulated in the environment to cause a serious environmental problem, which is urgently needed to develop novel treatments or control technology. The biodegradation of plastics has gained great attention due to the advantages of green and safe characteristics. Microorganisms play a vital role in the biodegradation of plastics, including environmental microbes (*in vitro*) and gut microbes of insects (*in vivo*). Microbial degradation in environmental conditions *in vitro* is extremely slow for major plastics at degradation rates on the basis of a month or even a year time, but recent discoveries show that the fast biodegradation of specific plastics, such as PS, PE, and PUR, in some invertebrates, especially insects, could be enhanced at rates on basis of hours; the biodegradation in insects is likely to be gut microbial-dependent or synergetic bioreactions in animal digestive systems. This review comprehensively summarizes the latest 7-year (2016–2022) publications on plastic biodegradation by insects and microorganisms, elucidates the mechanism of plastic degradation in insects and environmental microbes, and highlights the cutting-edge perspectives for the potential applications of plastic biodegradation.

## Introduction

Plastics are flexible materials mainly composed of long polymer chains with superior chemical stability and mechanical properties, which are widely used in the automotive industry, agriculture, construction, packaging, and textiles (Andrady and Neal, 2009; Andrady, 2011; Dris et al., 2015). The largest market for plastics is packaging, which has accelerated its growth because of disposable containers (Geyer et al., 2017). Plastics can be divided into degradable and non-degradable ones based on their degradability in natural environments (Brodhagen et al., 2015; Major et al., 2016). Conventional plastics, including polyethylene (PE), polystyrene (PS), polypropylene (PP), polyvinyl chloride (PVC), polyethylene terephthalate (PET), polyurethane (PUR), and other polymer compounds, have an extremely slow degradation rate in the environments (Wang et al., 2016). According to the European plastics reports of production, demand, and waste data (https://www.plasticseurope.org/en/resources/publications/4312-plastics-facts-2020), global plastic production reached nearly 370 million tons in 2019, which is 247 times more than that of the 1950s. When plastics were invented more than 100 years ago, they were regarded as safe and harmless synthetic organic polymers (Baekeland, 1909). However, plastics are commonly accumulated and distributed in the environment because of their difficult degradation characteristic. An estimated 6.3 billion tons of plastics have been dumped since 1950 (Geyer et al., 2017). In the middle-income and high-income countries, the proportion of plastics in municipal solid waste increased from less than 1% in 1960 to more than 10% in 2005 (Jambeck et al., 2015), which raised huge concerns about plastic pollution, especially in the oceans (Worm et al., 2017).

Plastics can be degraded in natural environments by mechanical, photochemical, thermal, and biochemical mechanisms (Gewert et al., 2015). Photochemistry is the most efficient chemical degradation pathway for plastics in nature. Thermal oxidation proceed slowly at ambient temperature (Gewert et al., 2015), but with the increase in temperature, the thermal oxidation efficiency will also increase with the rising temperature. Taking advantage of the complex enzyme system, microorganisms can effectively degrade plastic polymers and obtain energy from them (Chen et al., 2020), which is considered a more environmentally friendly approach to eliminating plastic waste (Chen et al., 2020), but the microbial degradation rate is extremely slow and it is questionable whether the energy obtained from plastic degradation could support microbial activities, especially growth.

In 1975, a bacterium, *Flavobacterium*, was discovered to break down nylon in wastewater pools from a nylon factory (Kinoshita et al., 1975). Later, an increasing number of microorganisms had been found to degrade polymers from the natural environments, including soil, seawater, sludge, and compost (Jones et al., 1974; Albertsson et al., 1978; Albertsson and Karlsson, 1988; Pegram and Andrady, 1989; Otake et al., 1995; Ohtake et al., 1998; Artham et al., 2009). The story of insects and plastics begins with consumers' complaints that chocolate-based consumable packaging was being eaten by insects (Terence, 1997). The degradation capacity of insects was reported based on the observation of insects destroying and eating plastic packaging materials. Nowadays, scientists have screened many environmental microbes (*in vitro*) and gut microbes of insects (*in vivo*) to degrade plastics (Skariyachan et al., 2016). Thus, this review aims to timely provide new insights and solutions for the environmental pollution problems, which focused on the relationships between plastic biodegradation by insects and environmental microbes.

## Methodology

References were retrieved from ISI Web of Science (http://www.isiknowledge.com), Wiley (https://onlinelibrary.wiley.com/), PubMed (http://www.ncbi.nlm.nih.gov/pubmed), Scopus (http://www.scopus.com/), Springer Link (https://link.springer.com/), and ScienceDirect (http://www.sciencedirect.com/) databases. The following keywords and strings were searched: (synthetic OR non-biodegradable), (plastics particles OR microplastics/nanoplastics), (source OR fate), (exposure pathway OR way), (biodegradation OR plastic degradation), (microorganisms OR microbial OR microbes), (actinomycetes OR actinomycetal), (algae OR algal), (bacteria OR bacterial), (fungi OR fungus OR fungal), (enzyme OR enzymatic OR biocatalysts), (factors), (enzyme OR biocatalysts), and (mechanism OR steps OR processes). Published articles on enzymatic and microbial degradation of non-biodegradable plastics were filtered. References on the degradation of biodegradable plastics were excluded, and the aim was focused on publications within the latest 12 years (2010–2022), except where there is a lack of recent literature on the subject. Also, the keywords of “Plastics” and “Biodegradation” were used for searching back to 1970. Three independent searches were performed, and the consistency of selected papers was confirmed. Data from the search results were reviewed, analyzed, categorized, and expressed in suitable sections to cover the scope of this review.

## Biodegradation of plastics

**Figure 1:**
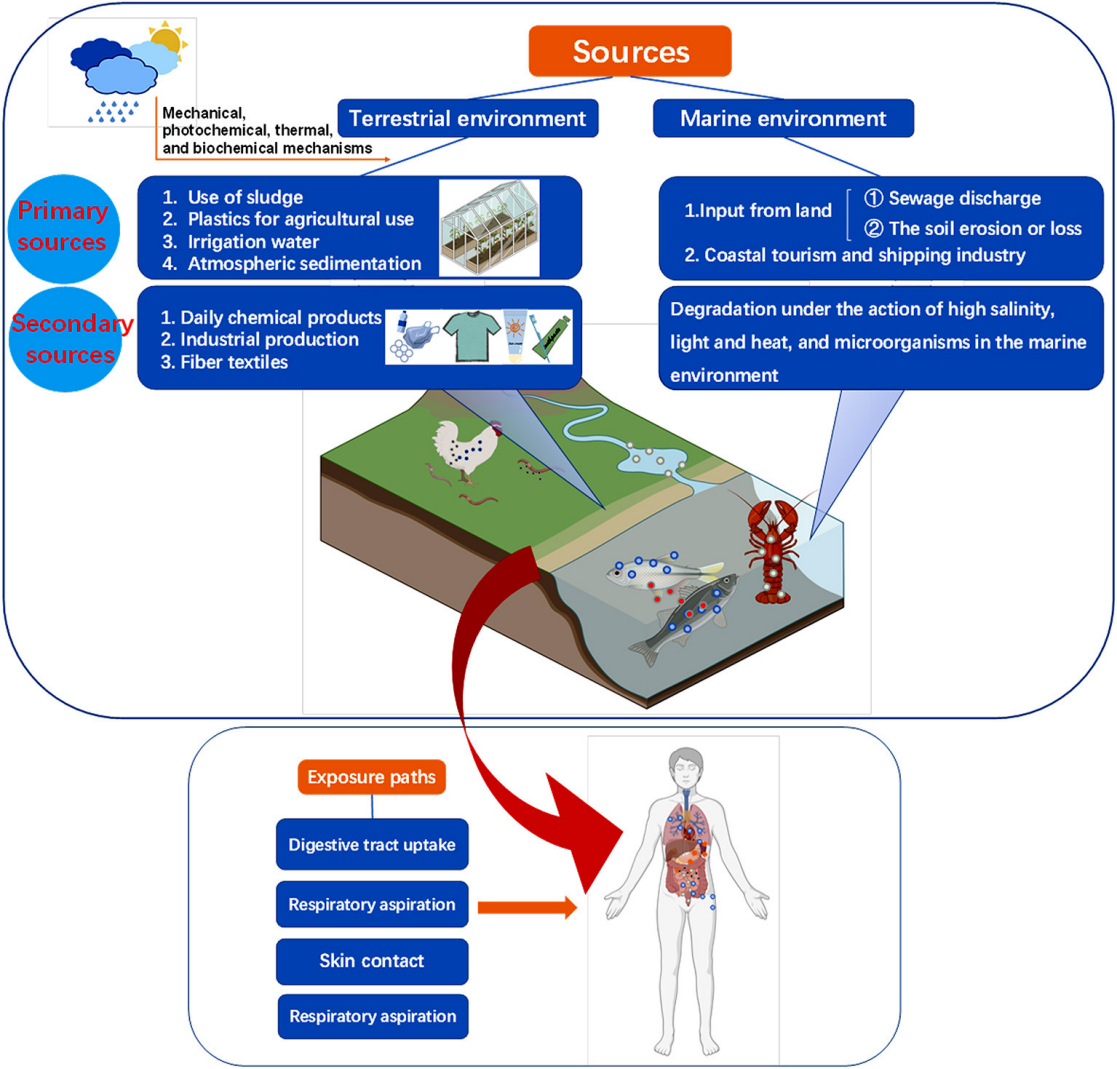
Sources and exposure paths of micro (nano) plastics from environments to the human body.

Usually, the degradation mechanisms of plastics include photooxidation degradation, catalytic degradation, ozone-induced degradation, thermal degradation, mechanical degradation, and biodegradation, among which the final products of biodegradation are CO_2_ and water, which has the advantages of green environmental protection and low energy consumption (Lu et al., 2013). The plastics in the environments can be degraded into microplastics (MPs) or nanoplastics (NPs) under the action of weathering, cracking, and decomposition, involved in the physical, chemical, and biological processes (Luo et al., 2018). Plastic waste seems to be gone, but it exists everywhere as MPs or NPs, which possesses severe damage potential for health risks (Figure 1). Previous studies reported that specific organisms can turn plastics into small fragments or even NPs (Mateos-Cárdenas et al., 2020). For example, Antarctic krill can turn MPs into NPs through digestive fragmentation (Dawson et al., 2018), and it is a bad sign because NPs are more problematic than MPs based on toxicology. Currently, researchers have found that many bacterial and fungal strains can degrade MPs under laboratory conditions and in the environment (Ru et al., 2020; Yuan et al., 2020). Saini et al. reported the possible biodegradation approaches and techniques for MPs (Miri et al., 2022). All possible approaches that he mentioned include microbial degradation of primary or secondary MPs by using microorganisms or key enzymes (Miri et al., 2022). Plastic-degrading microbes and their degrading plastic types are shown in Table 1. Moreover, the biodegradation of plastics was also summarized, and their inner mechanisms by insects and environmental microbes were highlighted, which will be of great benefit for researchers to investigate safe and efficient treatments for plastic wastes.

**Table 1:** Plastic-degrading microbes.

**Microbes**	**Types of plastics**	**References**
**Bacteria**		
*Acinetobacter baumannii*	PE	Pramila and Ramesh, 2015
*Anoxybacillus rupiensis*	Nylon	Mahdi et al., 2016
*Achromobacter denitrificans*	PE	Ambika et al., 2015
*Bacillus cereus*	PE	Ambika et al., 2015
*Bacillus* spp. *Stenotrophomonas pavanii*	PE	Muhonja et al., 2018
*Bacillus simplex*	PE	Huerta Lwanga et al., 2018
*Bacillus amyloliquefaciens*	PE	Novotný et al., 2018
*Bacillus* sp.	PE, PVC	Huerta Lwanga et al., 2018; Novotný et al., 2018; Park and Kim, 2019
*Enterobacter* sp.	PE	Ren et al., 2019
*Lysinibacillus* sp. *Salinibacterium* sp.	PE	Syranidou et al., 2017
*Ideonella sakaiensis*	PET	Sudhakar et al., 2008; Yoshida et al., 2016
*Lysinibacillus fusiformis*	PE	Ambika et al., 2015
*Paenibacillus* sp.	PP	Park and Kim, 2019
*Brevibacillus borstelensis*	PE, PET	Hadad et al., 2005; Calabia and Tokiwa, 2006; Muhonja et al., 2018
*Bacillus cereus* *Pseudomonas putida*	PE	Muhonja et al., 2018
*Pseudomonas fluorescens* B-22	PVC	Danko et al., 2004; Gilan et al., 2004
*Pseudomonas* sp.	PS	Umamaheswari and Subramani, 2017
*Pseudomonas aestusnigri*	PET	Bollinger et al., 2020
*Pseudomonas protegens*	PU	Hung et al., 2016
**Fungi**		
*Aspergillus fumigatus* *Aspergillus oryzae* *Aspergillus nidulans*	PE	Muhonja et al., 2018
*Aspergillus tubingensis*	PE	Sangeetha Devi et al., 2015
*Aspergillus flavus*	PVC	Zhang et al., 2020
*Aspergillus nomius*	PE	Abraham et al., 2017
*Aspergillus terreus* *Aspergillus sydowii*	PE	Sangale et al., 2019
*Aspergillus niger* *Penicillium pinophilum*	PE	Volke-Sepúlveda et al., 2002
*Cephalosporium* sp. *Mucor* spp.	PS	Chaudhary and Vijayakumar, 2020
*Curvularia senegalensis* *Fusarium solani*	PUR	Howard, 2002
*Cladosporium cladosporioides*	PU	Álvarez-Barragán et al., 2016
*Cochliobolus* sp.	PVC	Sumathi et al., 2016
*Engyodontium album* *Phanerochaete chrysosporium*	PP	Jeyakumar et al., 2013
*Penicillium simplicissimum*	PE	Yamada-Onodera et al., 2001
*Pestalotiopsis microspore*	PUR	Russell et al., 2011
*Zalerion maritimum*	PE	Paço et al., 2017
**Actinomycete**		
*Streptomyces scabies*	PE	Jabloune et al., 2020
*Streptomyces sp*.	PET, PE	Abraham et al., 2017; Farzi et al., 2017, 2019
*Streptomyces species* (1) and (2) *Pseudonocardia* *Actinoplanes* *Sporichthya*	PE	Sathya et al., 2012
*Actinomadura miaoliensis* sp. nov.	PE	Tseng et al., 2009
*Nocardiopsis sp*.	PE	Singh and Sedhuraman, 2015
**Algae**		
*Scenedesmus dimorphus* *Anabaena spiroides* *Navicula pupula*	PE	Gopal, 2017
*Spirulina sp*.	PET, PP	Khoironi et al., 2019

### Biodegradation of plastics by insects and other invertebrate

Currently, many species of insects were reported to degrade plastics (Table 2), including mealworms (larvae of *Tenebrio molitor*) (Yang et al., 2018), superworms (larvae of *Zophobas atratus*) (Peng et al., 2020b), and greater wax moth larvae (*Galleria mellonella L*) (Jiang et al., 2021b; Wang S. et al., 2022). Notably, *Tenebrio molitor* exhibited amazing degradation efficiency and possessed a wide selection of plastic types. In a recent study in 2022, the efficiency of mealworms to degrade polyester-PU foam was as high as 67% (Liu et al., 2022). Currently, the widely reported types of plastics degraded by *Tenebrio molitor* included PE (Brandon et al., 2018), PS (Brandon et al., 2021), Polyester PU (Liu et al., 2022), and PVC (Peng et al., 2020a). *Tenebrio molitor* larvae not only degrade low-density polyethylene (LDPE) but also linear low-density polyethylene (LLDPE) and high-density polyethylene (HDPE). The depolymerization capability was influenced by plastic type, molecular weight, and branching number (Yang et al., 2022). Yang et al. found that the yellow mealworms can completely degrade PS into CO_2_ and assimilate it into their biomass (Yang et al., 2015). *Zophobas atratus* and *Tenebrio obscurus* have been reported to have a higher degradation capability for PS than yellow mealworms (Peng et al., 2019; Yang Y. et al., 2020). *Zophobas atratus* larvae can degrade PS and PE but do not generate NPs in their frass (Peng et al., 2020b, 2022), implying that plastic biodegradation by insects is a more environmentally friendly option. *Tribolium castaneum* was also proven to degrade PS, and *Acinetobacter* from the larvae of *Tribolium castaneum* was recently isolated (Wang et al., 2020). *Plodia interpunctella* can chew PE plastic, but the degradation of plastic is caused by *Enterobacter asburiae* and *Bacillus* in the intestinal tract (Graham Bowditch, 1997). The biodegradation of plastic polymer has also been verified by other invertebrates, including lesser waxworm (*Achroia grisella*) (Kundungal et al., 2019), confused flour beetle (*Tribolium confusum*) (Kundungal et al., 2019), land snail (*Achatina fulica*), and other invertebrates (Song et al., 2020). Many studies reported the potential use of earthworms to enhance the decay of biodegradable plastics (Sanchez-Hernandez et al., 2020; Christyraj et al., 2022). Recent studies suggest that earthworms, particularly anecic and endogeic species, may facilitate plastic biodegradation directly and indirectly *via* their strong impact on soil microbial properties and the intense soil bioturbation (Sanchez-Hernandez et al., 2020; Wang L. et al., 2022). Termites are the major soil insects that can also degrade plastics using their gut microbiota (López-Naranjo et al., 2013; Kumar et al., 2022), but more evidence is needed to be provided.

**Table 2:** The confirmed plastic-degrading insects and their ability to degrade diverse plastic materials.

**Insect species**	**Types of plastic**	**Degradation efficiency**	**Mechanisms**	**References**
*Tenebrio molitor*	PE, PS	49.0 ± 1.4% loss of PE and PS weight for 32 days	Gut microbiome- *Citrobacter* sp. and *Kosakonia* sp.	Brandon et al., 2018
	PS	/	Gut Microbiome- eight unique bacterial species	Brandon et al., 2021
	Polyether-PU foam	67% loss of PE-PU foam for 35 days	Gut Microbiome- the families *Enterobacteriaceae* and *Streptococcaceae*	Liu et al., 2022
	PE	1.818 g PE of loss on the 58th day	Gut microbiome	Bulak et al., 2021
	PS	0.07 mg PE/larvae/day	Gut Microbiome- *Enterococcus, Enterobacteriaceae, Escherichia-Shigell*, and *Lactococcus*.	Jiang et al., 2021a
	PS	22.0 ± 0.5 g PS loss in 2 weeks	*Cronobacter sakazakii* and *Lactococcus garvieae*	Bae et al., 2021
	PVC	65.4% loss of ingested PVC for 16 days	Gut microbiome	Peng et al., 2020a
*Zophobas atratus*	PS foam	36.7% loss of PS weight for 28 days	Gut microbiota	Yang et al., 2020
	PS	/	Gut Microbiome-*Pseudomonas* sp. EDB1, *Bacillus* sp. EDA4 and *Brevibacterium* sp. EDX	Arunrattiyakorn et al., 2022
	PS	2.78 mg PS/larvae/day	Gut Microbiome-*Enterococcus, Enterobacteriaceae, Kluyvera*, and *Lactococcus NDa*	Jiang et al., 2021b
	PS, LDPE	43.3 ± 1.5 mg PS/100 larvae per day, 52.9 ± 3.1 mg LDPE/100 larvae per day	Gut microbiota and microbial functional enzymes	Peng et al., 2022
	LDPE, EPS	58.7 ± 1.8 mg/100 larvae per day, 61.5 ± 1.6 mg EPS/100 larvae per day	Gut microbiota	Peng et al., 2020b
*Galleria mellonella*	PE, PS	0.88 and 1.95 g loss of PE and PS weight for 21days	Intestinal bacteria- *Bacillus* and *Serratia*	Lou et al., 2020
	LDPE	/	Gut Microbiome-*Acinetobacter, Cloacibacterium, Corynebacterium, Curvibacter, Enhydrobacter* and *Staphylococcus genera*	Latour et al., 2021
	LDPE	/	Gut microbiome	Réjasse et al., 2021
	PS	/	Gut microbiota	Wang et al., 2022
	PS	12.97 ± 1.05% loss weight of PS for 30 days	Intestinal bacteria-*Massilia* sp. FS1903	Jiang et al., 2021b
*Plodia interpunctella*	PE	6.1 ± 0.3% and 10.7 ± 0.2% loss of PE weight for 28 days	Two bacterial strains-*Enterobacter asburiae* YT1 and *Bacillus* sp. YP1	Yang et al., 2014
	PE	15.87% loss of PE weight for 60 days	*Meyerozyma guilliermondii* ZJC1 (MgZJC1) and *Serratia marcescens* ZJC2 (SmZJC2)	Lou et al., 2022
*Tribolium castaneum*	PS	12.14% loss of mass weight and 13%/25% (Mw/Mn) reduction of molecular weight for 60 days	An intestinal bacterium- *Acinetobacter* bacterium	Wang et al., 2020
*Tenebrio obscurus*	PS	32.44 ± 0.51 mg/100 larvae per day	Intestinal bacteria- *Enterobacteriaceae, Spiroplasmataceae*, and *Enterococcaceae*	Peng et al., 2019
*Tribolium confusum*	PS, PE, and EVA (Ethyl vinyl acetate)	51.92, 46.84, and 2.9% loss of PS, PE, and EVA, respectively, for 30 days	/	Abdulhay, 2020
*Achroia grisella*	HDPE (high-density polyethylene)	Loss weight of PE- (43.3 ± 1.6%) and PE + wax (69.6 ± 3.2%) for 8 days	/	Kundungal et al., 2019
*Spodoptera frugiperda*	PVC	19.57% loss of PVC weight for	Intestinal bacterium -Strain EMBL-1	Zhu et al., 2022
*Alphitobius diaperinus*	PS	/	Intestinal bacteria- *Pseudomonas* sp. 2 m/c	Cucini et al., 2022
*Uloma* sp.	PS	37.14 mg of PS per day per 100 larvae	Gut microbiota	Kundungal et al., 2021
*Corcyra cephalonica* (Stainton)	LDPE	Weight loss: without antibiotic feeding - 25% with antibiotic feeding - 21%	Gut microbiota	Kesti and Sharana, 2019
*Plesiophthalmus davidis*	PS	34.27 ± 4.04 mg PS loss/larva	Gut microbiota	Woo et al., 2020

Due to the different chemical properties of various plastics, the biodegradability in insects is also diverse. *Galleria mellonella* L. degraded polyethylene (PE) faster than polystyrene (PS) (Lou et al., 2020). Previous studies validated that the yellow mealworms preferred to eat the mixtures of plastics and nutrition to achieve better degradability of plastics (Brandon et al., 2018; Kundungal et al., 2019). A double degradation rate of PS was found when *Tenebrio molitor* larvae were fed with mixed PS and bran (Brandon et al., 2018). Beeswax can increase the species richness and evenness of the intestinal microbiome in PE-fed larvae (Lou et al., 2020). A continual diet of PS with supplemental nutrition enables better growth and enhanced PS degradation by the beetle larvae, which is similar to lesser waxworms. The ability of *Uloma* sp. larvae to degrade PS suggests the ubiquitous phenomena of plastic degradation among the beeswax-eating species (Kundungal et al., 2021b). The degradation mechanism is probably involved in those diets with high nutrients that increase the diversity of the intestinal microbiome in worms. Therefore, excellent organisms should be discovered to degrade target plastics, and the best diet formula for blending plastics and nutrition needs to be established. The phyla Actinobacteria (*Microbacterium awajiense, Rhodococcus jostii, Mycobacterium vanbaalenii*, and *Streptomyces fulvissimus*) and Firmicutes (*Bacillus simplex and Bacillus* sp.) isolated from *Lumbricus terrestris'* gut have been proven to degrade LDPE-MPS with high efficiency-−60% (Huerta Lwanga et al., 2018). Due to different invertebrate species, plastic materials, and evaluation methods, it is difficult to simply describe the differences in the degradation rates of various insects, but specific degradation efficiency data are summarized in Table 2. In addition, except for the invertebrates that confirmed their capabilities of plastic biodegradation, other invertebrates were also reported to eat plastics (Table 3), but their degradation abilities need further studies.

**Table 3:** Reported plastic-eating insects and the corresponding plastic types.

**Insect species**	**Types of plastic**	**References**
*Ephestia cautella*	PVC, PP	Graham Bowditch, 1997
*Rhyzopertha dominica*	PP, PE, PEST	Graham Bowditch, 1997
*Lasioderma serricorne*	PP, PE, PEST	Riudavets et al., 2007
*Sitophilus oryzae*	PP, PE, PEST	Riudavets et al., 2007
*Oryzaephilus surinamensis*	PE	Shukla et al., 1993
*Callosobruchus maculates*	PE	Shukla et al., 1993
*Stegobium paniceum*	PS	Davidson, 2012

### Mechanism of plastic degradation by insects

The process of degrading plastics by insects can be divided into five stages based on relevant studies: (1) Plastics are physically chewed by mouthparts and enter the intestinal tract; (2) microbes in the gut adhere to and erode plastic; (3) the plastic is depolymerized into oligomer fragments by oxidation or hydrolysis of enzymes, which are provided by both host and gut microbiome; (4) the host provides bioemulsifying agents enhancing the effectiveness of microbial and host enzymes to attack polymers; (5) the bonds of oligomers are broken to form fatty acids; and (6) fatty acids are decomposed *via* insect biological metabolism. To seek efficient approaches for plastic biodegradation, the function of intestinal microbiota in insects should be considered. A previous study reported that yellow mealworms lost the ability to degrade PS after inhibiting intestinal bacterial activity with antibiotics, implying that intestinal bacteria play a key role in plastic biodegradation (Yang et al., 2015). The biofilm was formed by the isolated strain YT2 on PS film after a 28-day incubation, and obvious pits and cavities were observed on PS film surfaces, accompanied by the decreasing hydrophobicity and the formation of C–O polar groups. Suspension culture of strain YT2 could degrade 7.4 ± 0.4% of the PS pieces after a 60-day incubation. The molecular weight of the residual PS pieces was lower, and water-soluble intermediates were released, implying the vital effect of mealworm gut bacteria on PS biodegradation and mineralization (Yang et al., 2015). The biodegradation of PP by superworms and yellow mealworms *via* gut-microbe-dependent depolymerization was also confirmed (Yang S. et al., 2020). Two gut bacteria for PE biodegradation, *Bacillus sp*. YP1 and *Enterobacter asburiae* YT1, were isolated from waxworms (Yang et al., 2014). Moreover, the efficiency of PS biodegradation and mineralization *in vitro* was much lower than that *in vivo*, suggesting that the accelerated degradation of plastic in insects may be a complex process depending on both the microbiome and the host (Yang et al., 2015). Notably, the physicochemical “treatments” of chewing, ingestion, mixing with intestinal contents, and enzymes secreted by worms may be critical for the rapid degradation of PS *in vivo* (Yang et al., 2015). Brandon et al. provided evidence that *T. molitor* secreted one or more emulsifying factor(s) (30–100 kDa) to mediate plastic bioavailability. They also demonstrated that the insect gut microbiome secreted emulsifying factor(s) (< 30 kDa) that enhanced respiration on polystyrene (PS) (Brandon et al., 2021).

**Figure 2:**
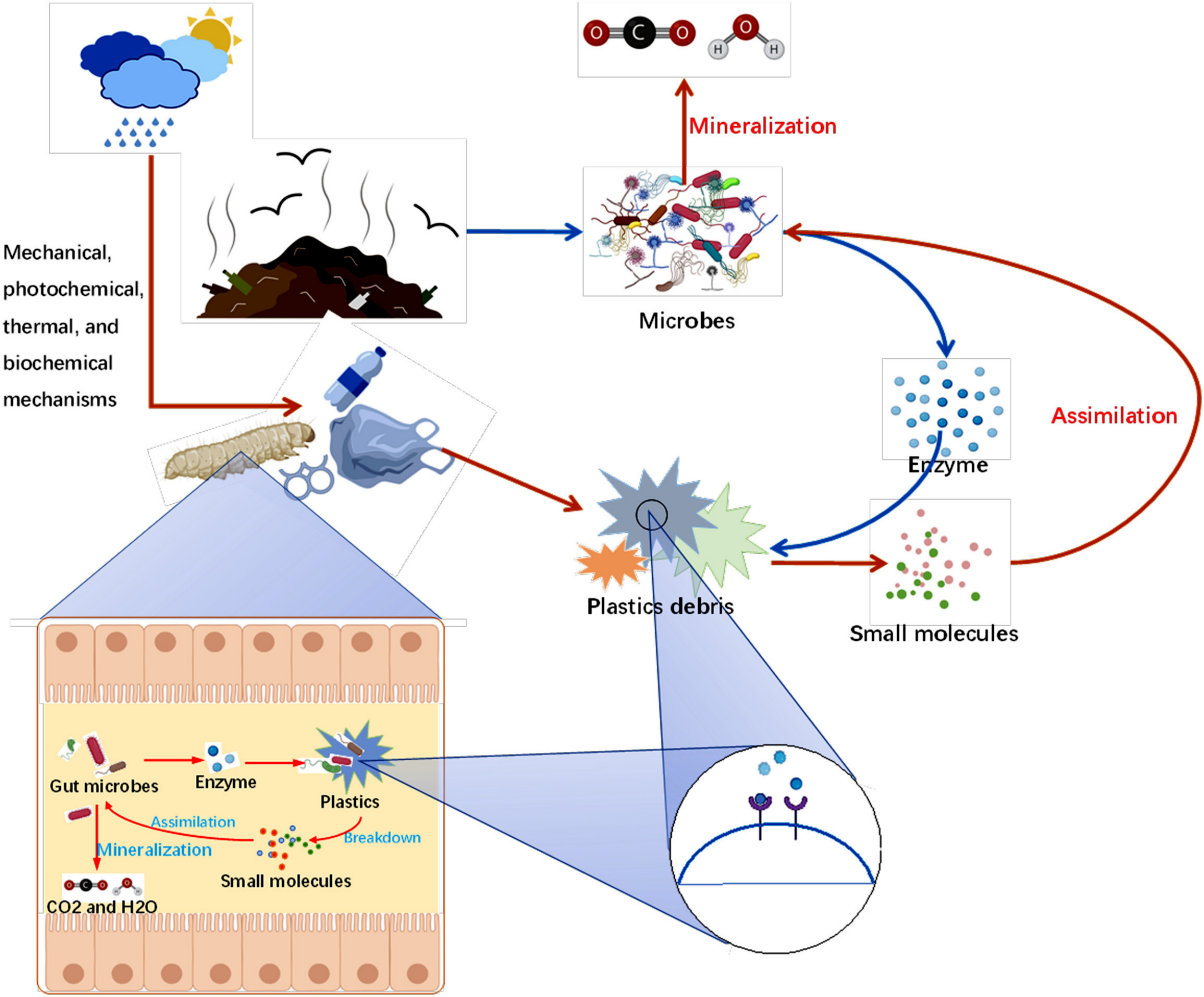
Biodegradation of plastics by insects and environmental microbes.

In addition to the insects' gut bacteria, gut fungi also can degrade plastics. Recently, Zhang et al. isolated a PE-degrading fungus, *Aspergillus flavus*, from the intestine of a Wax moth larva (*Galleria mellonella*), which can degrade HDPE MP to low molecular weight MP after 28 days of culture (Zhang et al., 2020). Moreover, two Laccase-like multicopper oxidase (LMCOS) genes, *Afla_053930* and *Afla_006190*, are upregulated during the degradation process, which is related to PE degradation (Zhang et al., 2020). Enzymes secreted by bacteria and fungi from insects may be the direct reason for plastic degradation. Plastic polymers are mainly depolymerized by extracellular enzymes into short chains or small molecules and then transported to cells for complete oxidation (Amobonye et al., 2021). Bacteria can produce many extracellular enzymes to degrade plastic macromolecules, such as lipases, depolymerase, esterase, proteinase K, cutinase, urease, and dehydrase (Shahnawaz et al., 2019; Taniguchi et al., 2019). For example, the degradation of PET is closely related to PET hydrolase, which can change the polymer chain or ring structure to enhance the efficiency of enzymatic hydrolysis (Kawai et al., 2019). As shown in Figure 2, the potential progress of plastic biodegradation by insects was summarized based on the above references and reports, which provided novel perspectives for the biodegradation treatment of different materials including PE, PS, PP, PET, and PUR.

### Degradation of plastics by environmental microbes

The importance of insect gut microbes for plastic biodegradation has been well documented earlier, and abundant plastic-degrading microbiota in the environment also play important roles. Recently, different actinomycetes, algae, bacteria, and fungi with the potential to biodegrade various plastic polymers have been investigated. To date, more than 56 species of bacteria and fungi belonging to 25 genera have been reported for polyethylene biodegradation, whose main sources are soil and landfills (Cassone et al., 2020; Kundungal et al., 2021a). *Bacillus, Pseudomonas, Streptomyces, Rhodococcus ruber*, and other bacteria were found to degrade PE (Park and Kim, 2019; Zhang et al., 2021). PET degradation by *Thermobifida fusca, Aspergillus Niger*, and *Bacillus subtilis* is recently reported (Barth et al., 2016). PUR degradation by *Curvularia senegalensis* and *Fusarium solani* (Khan et al., 2017) is also discovered. Moreover, the biodegradable bacteria from marine water, such as PE and PVC-degrading *Bacillus* sp., were recently isolated (Kumari et al., 2019). Actinomycetes including *Streptomyces, Rhodococcus ruber, Actinomadura* spp., and *Thermoactinomyces* have been isolated from different environments and confirmed to possess significant plastic biodegradative potentials (Auta et al., 2018; Jabloune et al., 2020; Amobonye et al., 2021). The hydrolytic enzymes they release are one of the main factors responsible for their growth on different plastic polymers and for degrading the high molecular weight compounds to low ones (Gohain et al., 2020). PET, PUR, p-nitrophenyl esters, keratin, rubber diesel, and different chemical additives are found to be degraded by actinomycetes (Singh and Sedhuraman, 2015; Gaytán et al., 2019; Jabloune et al., 2020).

Interestingly, algae, especially microalgae, also showed the ability to degrade plastic through the toxin systems or enzymes they secrete (Chia et al., 2020). The biological treatment of PE sheets with *Anabaena spiroides* (blue-green algae), *Navicula pupula* (diatom), and *Scenedesmus dimorphus* (green microalga) has been studied. After incubation at room temperature (27 ± 2°C) under light (12:12-h dark and light) for 1 month, *Anabaena spiroides* showed the most efficient degradation of plastics, which may degrade LDPE with an efficiency of 8.18% (Gopal, 2017). Moreover, when microalgae degrade plastic polymers, the process is involved in the reduction of activation energy to weaken the chemical bonds of PE polymers and consume polymers as a carbon source (Chia et al., 2020; Khoo et al., 2021; Soong et al., 2022). *Spirulina* sp. could biodegrade PET and PP, but the degradation efficiency was significantly lower, compared to bacteria and fungi (Khoironi et al., 2019). The reason may be that microalgae, unlike bacteria, use atmospheric CO_2_ as the sole carbon source and sunlight as the main energy source (Dineshbabu et al., 2020). Recently, *Phaeodactylum tricornutum* was reported for the high-efficiency biodegradation of PET due to its successfully engineered PETase from *Ideonella sakaiensis* (Moog et al., 2019).

However, the efficiency of plastic degradation by various microorganisms is relatively slow, which hinders the practical application of plastic biodegradation in the industry (Amobonye et al., 2021). Currently, no *in vitro* technique of plastic degradation fits industrial applications. Thus, genetically engineered microorganisms with delicate designs by integrating efficient plastic-degrading enzymes would be feasible for practical application.

### Mechanism of plastic degradation by environmental microbes

**Figure 3:**
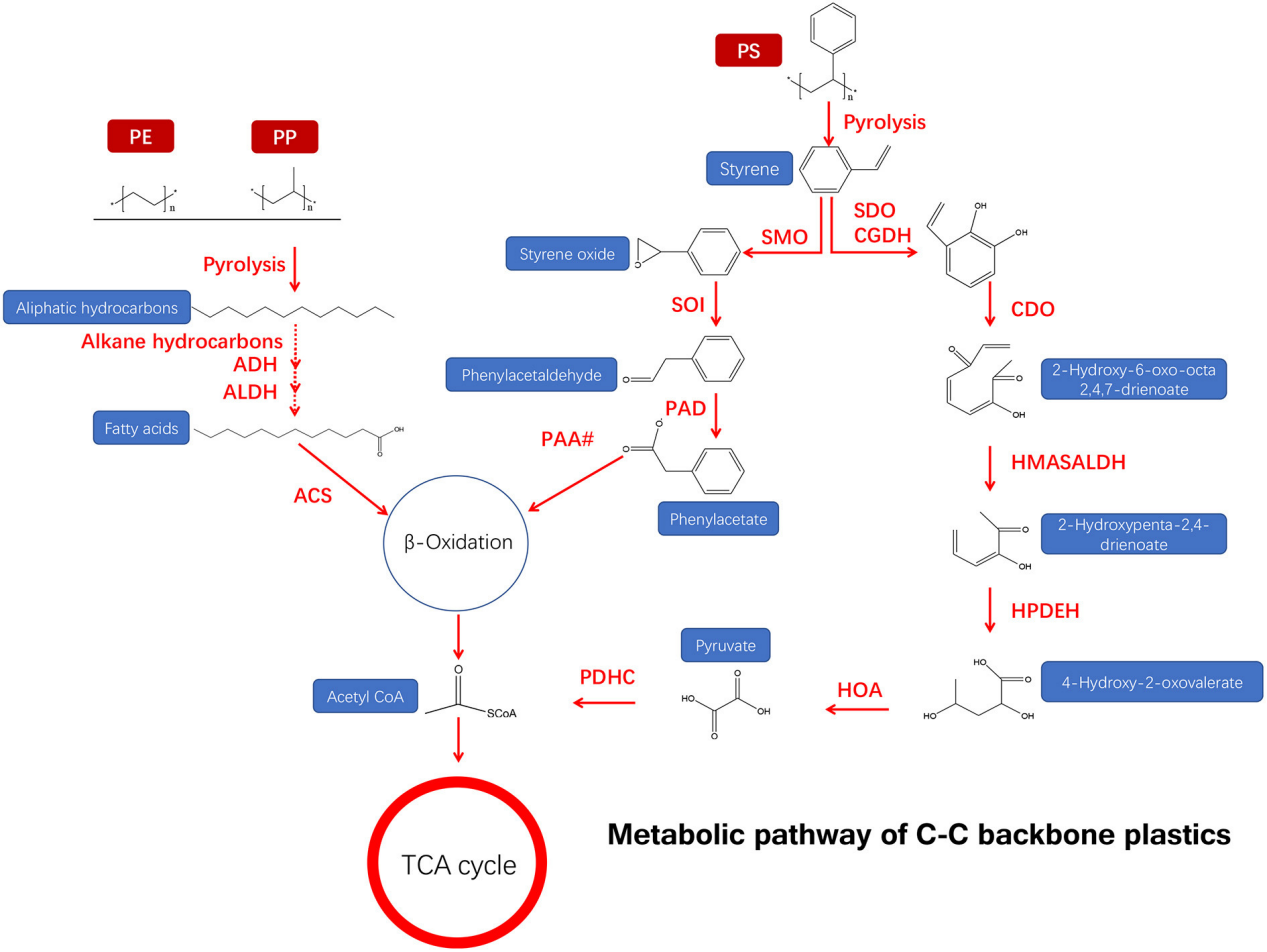
Metabolic pathway of a C–C backbone synthetic plastic material (adapted from previous studies Ru et al., 2020; Ali et al., 2021).

The primary determinant of biodegradable plastic polymers is the property of the bonds linking monomers together. Among the six major types of synthetic plastics (PE, PP, PS, PVC, PUR, and PET), the C–C backbones of PE, PP, PS, and PVC are highly recalcitrant (Figure 3), while PUR and PET with a hydrolyzable backbone are more vulnerable to enzymatic degradation (Chen et al., 2020). Plastic degradation by environmental microbes may be involved in the below steps: (1) Environmental microbes release hydrolase specifically binding to plastic surface receptors and then hydrolyze plastics to molecules; (2) those small molecules of acids or lipids generated from the previous process can enter the microorganism and participate in their physiological metabolic process and are further decomposed into water and carbon dioxide, releasing energy for cell growth (Crawford and Quinn, 2017; Zumstein et al., 2018). Thus, efficient plastic biodegradation can be achieved based on the degradation mechanism.

#### Microbial degradation of C–C bond plastics

For PE degradation, the biodegradation process usually involves bio-fragmentation of PE polymer by secreted enzymes, followed by bio-assimilation of small lytic fragments by microorganisms (Bonhomme et al., 2003; Montazer et al., 2019). Specifically, the –C–C– groups of a long-chain backbone of PE are oxidized into the –C=O– (carbonyl) group by the microbe, and the small aliphatic hydrocarbons can be transported directly into the cell for degradation (Albertsson et al., 1987, 1998; Desforges et al., 2015).

For PS degradation, the thickness and molecular weight of plastics are the main factors affecting the biodegradation rate (Krueger et al., 2015). Tischler et al. demonstrated that *Rhodococcus opacus* 1CP, a strain of actinobacterium, can mineralize styrene by styrene oxide *via* the aerobic phenylacetic acid (PAA) pathway (Tischler et al., 2009), indicating that styrene is degraded by a monooxygenase-catalyzed epoxidation of the vinyl side chain, and styrene oxide further converted it to phenylacetaldehyde with the help of an epoxystyrene isomerase (Tischler et al., 2009). This metabolic pathway is involved in multiple enzymes, including styrene monooxygenase (SMO), phenylacetaldehyde dehydrogenase (PAD), styrene oxide isomerase (SOI), and other enzymes of phenylacetate (PAA#) degradation accessing the tricarboxylic acid cycle (TCA) (Figure 3) (Tischler et al., 2009). The side-chain oxygenation pathway is very common for the aerobic degradation of styrene, which was reported in the proteobacteria genera *Pseudomonas* and *Xanthobacter* (O'leary et al., 2002). Moreover, phenylacetaldehyde may be further oxidized to PAA by the action of a phenylacetaldehyde dehydrogenase.

The degradation pathway of PVC is not the same as PE and PP because it contains Cl^−^. Oxidation of chlorinated hydrocarbons is much more difficult than PE and PP. For example, *T. molito* can mineralize PE, PP, and PS, but the mineralization of PVC is poor because most PVC is converted to chlorinated intermediates (Peng et al., 2020a). Currently, there are few studies on the mechanism of PVC biodegradation, and most of them just focus on screening certain bacteria that can degrade PVC. Because of the chemical stability and hydrophobicity of the C–C skeleton of PVC, there is no report on the enzymes directly involved in its degradation. The only enzyme found was laccase (Sumathi et al., 2016). Laccase (EC 1.10.3.2) is a kind of oxidoreductase with the ability to oxidize phenolic compounds, which has been widely used in the decomposition of lignin, phenolic substances, and toxic pollutants (Janusz et al., 2020). Sumathi et al. (2016) proved that laccase could break PVC double bonds and generate new C=O bonds, but the specific mechanism was unknown. Based on the degradation pathways of cellulose, lignin, and other macromolecules, it was speculated that oxygen-free radicals in laccase products might attack C–C bonds. The short chain produced by this reaction is degraded by other unknown enzymes, which can be used by microorganisms as a carbon source through the TCA cycle.

Both PVC and PP are highly hydrophobic and resilient to chemical abrasion (Shah et al., 2008), which makes it difficult to degrade them *via* microbial activity. Although several microbial strains were proven to own the ability to degrade PVC and PP (Sah et al., 2011; Jeyakumar et al., 2013), the essential degradation enzymes and the underlying degradation mechanism remain unknown. Therefore, the depolymerization of PVC and PP should be further studied.

#### Microbial degradation of hydrolyzable bond plastics

**Figure 4:**
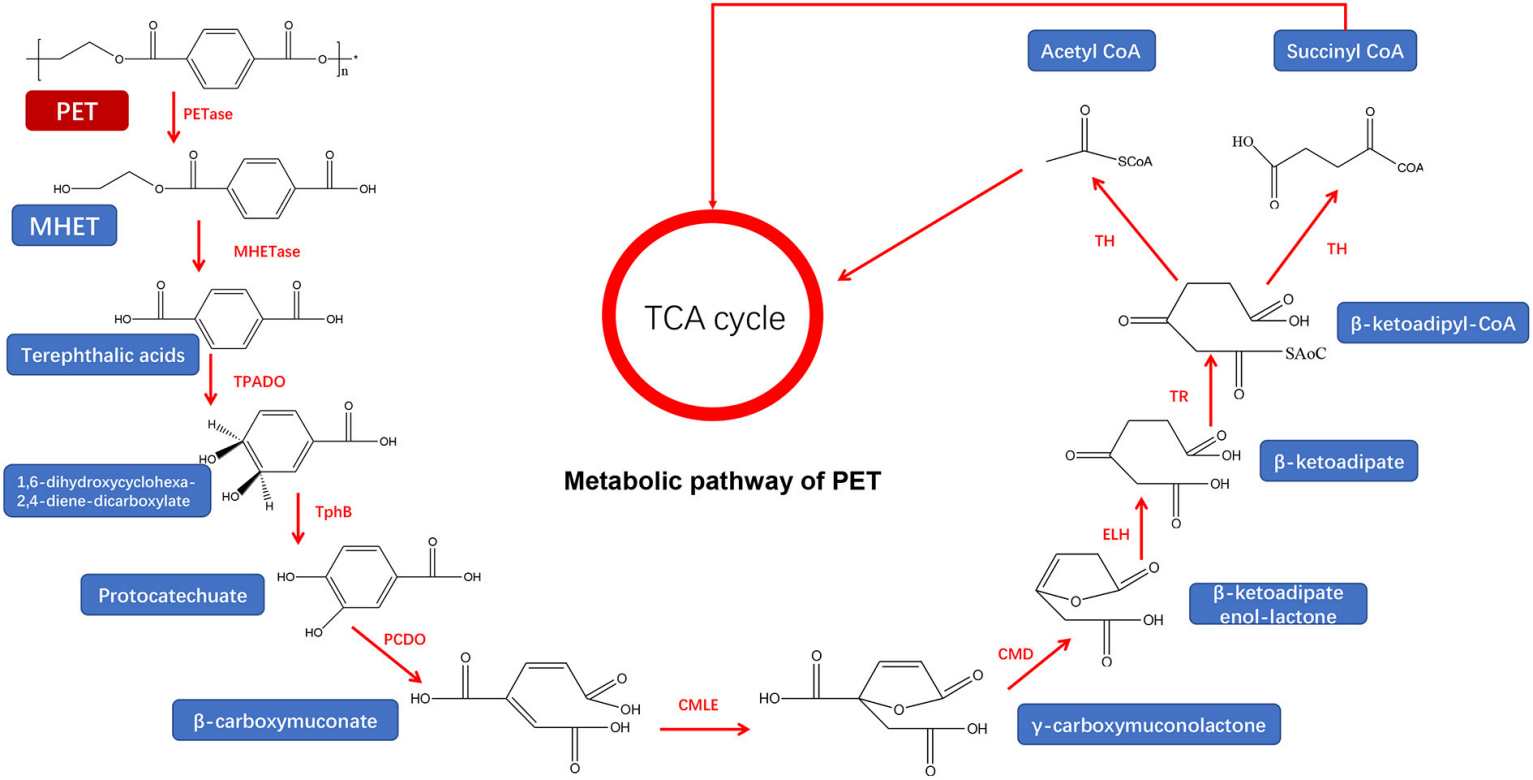
Metabolic pathway of PET (adapted from previous studies Yoshida et al., 2016; Ru et al., 2020).

For the ester-linked PET degradation, the PET and PET hydrolase can target the terminal or ring structure of the polymer chains for enzymatic hydrolysis, which increases the hydrophilicity of the PET and improves the subsequent enzymatic hydrolysis efficiency (Kawai et al., 2019). *Ideonella Sakaiensis 201-F6*, a bacterium from the genus *Ideonella*, was reported to degrade and assimilate PET (Yoshida et al., 2016) after the generation of PETase and MHETase that efficiently converts PET into environmentally friendly monomers, terephthalic acid, and ethylene glycol (Figure 4) (Yoshida et al., 2016). Notably, this PET hydrolase has 45–53% homology with actinomycete keratinase (Wei et al., 2019) but can completely degrade PET, compared to other PET hydrolases. However, the low stability of PETase limits its wide application. After enzymes digested the ester bond, PET is degraded into MHET. MHET can continue to be hydrolyzed into TPA and EG under the action of MHETase (Peng et al., 2019) and finally enter the tricarboxylic acid cycle (TCA cycle) (Ronkvist et al., 2009).

**Figure 5:**
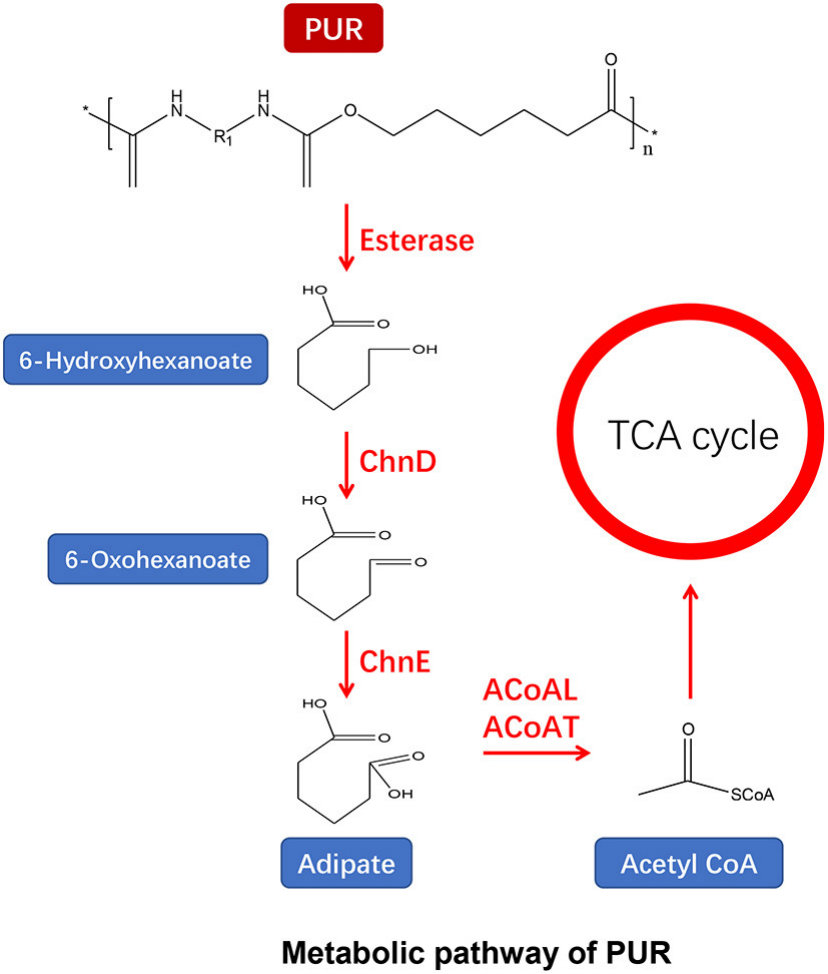
Metabolic pathway of PUR (adapted from previous studies Yoshida et al., 2016; Ru et al., 2020; Ali et al., 2021).

PUR linked by urethane bonds is composed of di- or polyisocyanate and polyols (Seymour and Kauffman, 1992). Previous studies have reported the enzymes degrading polyester PUR from bacteria (Shah and Green, 1994; Nakajima-Kambe et al., 1995; Howard and Blake, 1998; Stern and Howard, 2000; Howard et al., 2012; Schmidt et al., 2017) and fungi (Crabbe et al., 1994; Russell et al., 2011). PUR can be depolymerized by microbial ureases, esterases, and proteases for hydrolyzing urethane and ester bonds (Figure 5) (Howard, 2012; Loredo-Treviño et al., 2012; Cregut et al., 2013). It has been postulated that proteases can hydrolyze the amide and urethane bonds, while ureases may attack the urea linkages (Labow et al., 1996; Ruiz et al., 1999; Matsumiya et al., 2010). Esterases and proteases can hydrolyze the ester bonds as a major mechanism for PUR depolymerization (Wei and Zimmermann, 2017). According to their localization, PUR-degrading enzymes are divided into membrane-bound and secreted types. The membrane-bound enzymes directly adhere to the PUR surface and hydrolyze the urethane bond, resulting in releasing monomers of the PUR (Cregut et al., 2013). For insoluble PUR, many secretases are released for the degradation of PUR (Wei and Zimmermann, 2017).

## Future perspectives

The accumulations of plastics in environments and wild animals are serious and pose risks to human health *via* the food chain. Thus, the development of biodegradable plastics and the final degradation without toxicity will be urgently needed to solve the problem of white pollution. Compared to traditional methods, the biodegradation of plastics using insects and environmental microbes becomes a potential application in the industrial treatment of plastic waste, but the application of insects for plastic waste treatment is not practicable now. Both insect degradation and microbial degradation are basically environmental science. Enzymatic degradation of PET could have an application future based on cost-effectiveness. This review provides new insights and approaches to solving the problem of plastic pollution from the biodegradation aspects. Due to the different physical and chemical properties of plastics, the degradation efficiency of insects varies greatly. Different types of plastics may affect the growth and development of insects; thus, the degradation toxicity caused by insects should be considered (Sanchez-Hernandez, 2021). More studies on the biodegradable application of plastics by insects and environmental microbes are still recommended. In future, in-depth studies on the following aspects should be considered: (1) Based on the characteristics of plastic-degrading insects, more environmental and gut microbes with a strong degrading ability should be screened to enrich the plastic-degrading insect library. (2) To ensure the normal growth and reproduction of insects, the proportion of plastic in the food diet should be optimized to further improve their degradation efficiency on plastic. (3) With the help of protein engineering and synthetic biology technology, efficient and artificial synthetic microorganisms can be constructed by modifying the plastic-degrading enzymes and designing metabolic pathways. (4) Since human health and the ecological environment harms are inevitably caused by plastics, the in-depth toxicological analysis of plastic-degrading insects and their gut microorganisms should be performed to avoid toxicological risks. (5) And to eliminate the toxicity risks from the source, it is urgent to advocate the use of biodegradable plastics, especially in takeout, e-commerce, and other industries with the widespread use of disposable plastic products.

## Author contributions

X-GY and P-PW: investigation. Y-FY and P-PJ: resources. P-PW and W-GL: data analysis. P-PW and D-SP: writing—original draft preparation. D-SP: writing—revision. All authors have read and approved the manuscript.
