# Corrigendum: Plastic biodegradation by *in vitro* environmental microorganisms and *in vivo* gut microorganisms of insects

**DOI:** 10.3389/fmicb.2024.1444678

**Published:** 2024-07-08

**Authors:** Xian-Guang Yang, Ping-Ping Wen, Yi-Fan Yang, Pan-Pan Jia, Wei-Guo Li, De-Sheng Pei

**Affiliations:** ^1^State Key Laboratory Base of Cell Differentiation and Regulation, College of Life Science, Henan Normal University, Xinxiang, China; ^2^School of Public Health, Chongqing Medical University, Chongqing, China

**Keywords:** enzyme, gut microbes, insects, invertebrate, plastic biodegradation

In the published article, there was an error in a section title. Instead of **Biodegradation of plastics by insects**, it should be **Biodegradation of plastics by insects and other invertebrate**.

In the published article, there was an error in Tables 2, 3 as published. The heading **Insect species** in the first cell of the first column and row of Tables 2, 3 were incorrect, due to the fact that not all species listed in these tables are insects. For instance, *Achatina fulica* is not an insect but rather a mollusk belonging to the class *Gastropoda. Sphaeroma terebrans* is not an insect but rather an *Arthropoda* belonging to the class *Crustacea*.

The corrected Tables 2, 3 and their captions appear below.

**Table 2:** The confirmed plastic-degrading insects and their ability to degrade diverse plastic materials.

**Insect species**	**Types of plastic**	**Degradation efficiency**	**Mechanisms**	**References**
*Tenebrio molitor*	PE, PS	49.0 ± 1.4% loss of PE and PS weight for 32 days	Gut microbiome- *Citrobacter* sp. and *Kosakonia* sp.	Brandon et al., 2018
	PS	/	Gut Microbiome- eight unique bacterial species	Brandon et al., 2021
	Polyether-PU foam	67% loss of PE-PU foam for 35 days	Gut Microbiome- the families *Enterobacteriaceae* and *Streptococcaceae*	Liu et al., 2022
	PE	1.818 g PE of loss on the 58th day	Gut microbiome	Bulak et al., 2021
	PS	0.07 mg PE/larvae/day	Gut Microbiome- *Enterococcus, Enterobacteriaceae, Escherichia-Shigell*, and *Lactococcus*.	Jiang et al., 2021a
	PS	22.0 ± 0.5 g PS loss in 2 weeks	*Cronobacter sakazakii* and *Lactococcus garvieae*	Bae et al., 2021
	PVC	65.4% loss of ingested PVC for 16 days	Gut microbiome	Peng et al., 2020a
*Zophobas atratus*	PS foam	36.7% loss of PS weight for 28 days	Gut microbiota	Yang et al., 2020
	PS	/	Gut Microbiome-*Pseudomonas* sp. EDB1, *Bacillus* sp. EDA4 and *Brevibacterium* sp. EDX	Arunrattiyakorn et al., 2022
	PS	2.78 mg PS/larvae/day	Gut Microbiome-*Enterococcus, Enterobacteriaceae, Kluyvera*, and *Lactococcus NDa*	Jiang et al., 2021b
	PS, LDPE	43.3 ± 1.5 mg PS/100 larvae per day, 52.9 ± 3.1 mg LDPE/100 larvae per day	Gut microbiota and microbial functional enzymes	Peng et al., 2022
	LDPE, EPS	58.7 ± 1.8 mg/100 larvae per day, 61.5 ± 1.6 mg EPS/100 larvae per day	Gut microbiota	Peng et al., 2020b
*Galleria mellonella*	PE, PS	0.88 and 1.95 g loss of PE and PS weight for 21days	Intestinal bacteria- *Bacillus* and *Serratia*	Lou et al., 2020
	LDPE	/	Gut Microbiome-*Acinetobacter, Cloacibacterium, Corynebacterium, Curvibacter, Enhydrobacter* and *Staphylococcus genera*	Latour et al., 2021
	LDPE	/	Gut microbiome	Réjasse et al., 2021
	PS	/	Gut microbiota	Wang et al., 2022
	PS	12.97 ± 1.05% loss weight of PS for 30 days	Intestinal bacteria-*Massilia* sp. FS1903	Jiang et al., 2021b
*Plodia interpunctella*	PE	6.1 ± 0.3% and 10.7 ± 0.2% loss of PE weight for 28 days	Two bacterial strains-*Enterobacter asburiae* YT1 and *Bacillus* sp. YP1	Yang et al., 2014
	PE	15.87% loss of PE weight for 60 days	*Meyerozyma guilliermondii* ZJC1 (MgZJC1) and *Serratia marcescens* ZJC2 (SmZJC2)	Lou et al., 2022
*Tribolium castaneum*	PS	12.14% loss of mass weight and 13%/25% (Mw/Mn) reduction of molecular weight for 60 days	An intestinal bacterium- *Acinetobacter* bacterium	Wang et al., 2020
*Tenebrio obscurus*	PS	32.44 ± 0.51 mg/100 larvae per day	Intestinal bacteria- *Enterobacteriaceae, Spiroplasmataceae*, and *Enterococcaceae*	Peng et al., 2019
*Tribolium confusum*	PS, PE, and EVA (Ethyl vinyl acetate)	51.92, 46.84, and 2.9% loss of PS, PE, and EVA, respectively, for 30 days	/	Abdulhay, 2020
*Achroia grisella*	HDPE (high-density polyethylene)	Loss weight of PE- (43.3 ± 1.6%) and PE + wax (69.6 ± 3.2%) for 8 days	/	Kundungal et al., 2019
*Spodoptera frugiperda*	PVC	19.57% loss of PVC weight for	Intestinal bacterium -Strain EMBL-1	Zhu et al., 2022
*Alphitobius diaperinus*	PS	/	Intestinal bacteria- *Pseudomonas* sp. 2 m/c	Cucini et al., 2022
*Uloma* sp.	PS	37.14 mg of PS per day per 100 larvae	Gut microbiota	Kundungal et al., 2021
*Corcyra cephalonica* (Stainton)	LDPE	Weight loss: without antibiotic feeding - 25% with antibiotic feeding - 21%	Gut microbiota	Kesti and Sharana, 2019
*Plesiophthalmus davidis*	PS	34.27 ± 4.04 mg PS loss/larva	Gut microbiota	Woo et al., 2020

**Table 3:** The reported plastics-eating insects and the corresponding plastic types.

**Insect species**	**Types of plastic**	**References**
*Ephestia cautella*	PVC, PP	Graham Bowditch, 1997
*Rhyzopertha dominica*	PP, PE, PEST	Graham Bowditch, 1997
*Lasioderma serricorne*	PP, PE, PEST	Riudavets et al., 2007
*Sitophilus oryzae*	PP, PE, PEST	Riudavets et al., 2007
*Oryzaephilus surinamensis*	PE	Shukla et al., 1993
*Callosobruchus maculates*	PE	Shukla et al., 1993
*Stegobium paniceum*	PS	Davidson, 2012

In the published article, there was an error. A correction has been made to the **Biodegradation of plastics by insects**, paragraph two. The statement is incomplete, as the species enumerated in Tables 2, 3 are not entirely composed of insects. This sentence previously stated:

“Due to different insect species, plastic materials, and evaluation methods, it is difficult to simply describe the differences in the degradation rates of various insects, but specific degradation efficiency data are summarized in Table 2. In addition, except for the insects that confirmed their capabilities of plastic biodegradation, other insects were also reported to eat plastics (Table 3), but their degradation abilities need further studies”.

The corrected sentence appears below:

“Due to different invertebrate species, plastic materials, and evaluation methods, it is difficult to simply describe the differences in the degradation rates of various insects, but specific degradation efficiency data are summarized in Table 2. In addition, except for the invertebrates that confirmed their capabilities of plastic biodegradation, other invertebrates were also reported to eat plastics (Table 3), but their degradation abilities need further studies”.

The authors apologize for these errors and state that they do not change the scientific conclusions of the article in any way. The original article has been updated.
